# Ligand-induced activation of human TRPM2 requires the terminal ribose of ADPR and involves Arg1433 and Tyr1349

**DOI:** 10.1042/BCJ20170091

**Published:** 2017-06-16

**Authors:** Ralf Fliegert, Joanna M. Watt, Anja Schöbel, Monika D. Rozewitz, Christelle Moreau, Tanja Kirchberger, Mark P. Thomas, Wiebke Sick, Andrea C. Araujo, Angelika Harneit, Barry V.L. Potter, Andreas H. Guse

**Affiliations:** 1 The Calcium Signalling Group, Department of Biochemistry and Molecular Cell Biology, University Medical Center Hamburg-Eppendorf, Martinistrasse 52, 20246 Hamburg, Germany; 2 Wolfson Laboratory of Medicinal Chemistry, Department of Pharmacy and Pharmacology, University of Bath, Bath BA2 7AY, U.K.; 3 Medicinal Chemistry & Drug Discovery, Department of Pharmacology, University of Oxford, Mansfield Road, Oxford OX1 3QT, U.K.

**Keywords:** adenosine diphosphate ribose, protein structure, transient receptor potential channels

## Abstract

TRPM2 (transient receptor potential channel, subfamily melastatin, member 2) is a Ca^2+^-permeable non-selective cation channel activated by the binding of adenosine 5′-diphosphoribose (ADPR) to its cytoplasmic NUDT9H domain (NUDT9 homology domain). Activation of TRPM2 by ADPR downstream of oxidative stress has been implicated in the pathogenesis of many human diseases, rendering TRPM2 an attractive novel target for pharmacological intervention. However, the structural basis underlying this activation is largely unknown. Since ADP (adenosine 5′-diphosphate) alone did not activate or antagonize the channel, we used a chemical biology approach employing synthetic analogues to focus on the role of the ADPR terminal ribose. All novel ADPR derivatives modified in the terminal ribose, including that with the seemingly minor change of methylating the anomeric-OH, abolished agonist activity at TRPM2. Antagonist activity improved as the terminal substituent increasingly resembled the natural ribose, indicating that gating by ADPR might require specific interactions between hydroxyl groups of the terminal ribose and the NUDT9H domain. By mutating amino acid residues of the NUDT9H domain, predicted by modelling and docking to interact with the terminal ribose, we demonstrate that abrogating hydrogen bonding of the amino acids Arg1433 and Tyr1349 interferes with activation of the channel by ADPR. Taken together, using the complementary experimental approaches of chemical modification of the ligand and site-directed mutagenesis of TRPM2, we demonstrate that channel activation critically depends on hydrogen bonding of Arg1433 and Tyr1349 with the terminal ribose. Our findings allow for a more rational design of novel TRPM2 antagonists that may ultimately lead to compounds of therapeutic potential.

## Introduction

The presence of adenosine 5′-diphosphoribose (ADPR) in mammalian cells has been known for a long time ([1] and references therein), but was mainly considered to be a result of the metabolism of the redox coenzyme β-NAD^+^ (nicotinamide adenosine 5′-dinucleotide) or the Ca^2+^ mobilizing second messenger cyclic adenosine 5′-diphosphoribose [2], or degradation of either mono- or poly-ADP (adenosine 5′-diphosphate)-ribosylated proteins [3]. Activation of a non-selective cation channel of the melastatin subfamily of TRP (transient receptor potential) channels, TRPM2 (transient receptor potential channel, subfamily melastatin, member 2), by ADPR [4,5] has renewed interest in ADPR as a potential second messenger in its own right.

TRPM2 integrates a multitude of cellular and physical influences including intra- and extracellular Ca^2+^ concentration [6] and pH [7,8], temperature [9], tyrosine phosphorylation [10], and phosphorylation by PKCα [11]. While activation of TRPM2 by an increase in the free cytosolic Ca^2+^ concentration ([Ca^2+^]_i_) either in the absence of ADPR [12] or in the presence of low endogenous ADPR concentrations [13] has been discussed, the main mechanism of activation seems to be the binding of ADPR to the C-terminal NUDT9H domain of TRPM2. Increases in ADPR may occur either as a result of receptor stimulation [14], activation of the poly(ADP-ribosyl) polymerase (PARP)/poly(ADP-ribosyl) glycohydrolase (PARG) pathway induced by oxidative stress [15], activation of NAD glycohydrolase activity in mitochondria [16], or by activation of sirtuin 6 resulting in *O*-acetyl-ADPR, and upon further deacetylation, in ADPR [17]. Ca^2+^ entering via the activated channel subsequently sensitizes the channel to ADPR, resulting in a positive feedback loop [18].

The ADPR-binding NUDT9 homology (NUDT9H) domain of TRPM2 derives its name from, and shares 39% of its sequence with, the mitochondrial enzyme NUDT9 [4], a nucleotide pyrophosphatase from the family of Nudix proteins. Nudix proteins hydrolyze compounds in which a nucleotide diphosphate is linked to other organic structures. Some Nudix proteins (type II ADPRases) accept nucleotides like flavin adenine dinucleotide, coenzyme A, diadenosine polyphosphates, and NADH as substrates in addition to ADPR, whereas others (type I ADPRases) including NUDT9 show a pronounced specificity for ADPR [19]. To date, several crystal structures of ADPRases (mainly of microbial origin) have been resolved with high resolution [20–23] including human NUDT9 alone and in complex with either β-d-glucose or ribose 5′-phosphate [24]. However, no crystal structures of TRPM2 or the isolated NUDT9H domain of TRPM2 have yet been published. Thus, relatively little is known about the mode in which ADPR binds to TRPM2 and how this interaction ultimately results in activation of the channel.

Based on its similarity to NUDT9, the NUDT9H domain is suggested to bind ADPR in a cleft formed between the N*-*terminal and C-terminal domains. In human NUDT9, the C-terminus forms a helix-loop-helix structure containing the Nudix box, which is involved in the enzymatic activity by stabilization of the structure of the active site and co-ordination of magnesium ions [24]. Aberrations from the core of the consensus Nudix box (REFGEE in human NUDT9 vs. RILRQE in human TRPM2) are considered to be responsible for the largely reduced catalytic activity of TRPM2 [24]. In human TRPM2, mutations in the homologous area, such as replacement of Arg1404, abrogate channel activation by ADPR [16].

For NUDT9, while mutational analysis suggests that the Nudix box is involved in catalysis, substrate specificity is much more likely to be conferred by regions outside the core Nudix box lining the cleft between the N-terminal and C-terminal domains [24].

Many observations led us to hypothesize that the terminal ribose of ADPR might play a pivotal role in TRPM2 activation. During our earlier systematic evaluation of the structure–activity relationship of ADPR at TRPM2 using modified ADPR analogues [25], we observed that ADP alone had no significant agonist or antagonist activity at TRPM2. In the human NUDT9 crystal structure reported by Stoddard and colleagues [24], the product d-ribose 5-phosphate (d-rib-5-P) is fixed by 10 hydrogen bonds. These features are generally conserved in human TRPM2:His324 (His1488, amino acid residues in parenthesis indicate homologous residues in human TRPM2), Asp172 (not conserved), and two water molecules form hydrogen bonds with the C2 and C3 hydroxyl groups of the ribose, whereas Arg204 (not conserved), Arg273 (Arg1433), Tyr321 (Tyr1485), and Glu234 (Glu1409) form hydrogen bonds to the phosphate [24]. The active site of the ADPRase of the bifunctional nicotinamide mononucleotide adenylyltransferase (NMNAT)/ADPR pyrophosphatase from the cyanobacterium *Synechocystis* sp. shows a high degree of structural similarity to NUDT9 [26]. In this enzyme, the terminal ribose of ADPR interacts with the side chains of amino acids Arg280 (Arg1433) and His326 (His1488), which are conserved in TRPM2. The terminal ribose also interacts with Asp205 and Arg277 that are not conserved. However, the corresponding residues in TRPM2 (Tyr1349 and Asp1430) might, as suggested by Huang et al. [26], also be able to form hydrogen bonds to the terminal ribose. The interactions described above may contribute to the high substrate specificity of this enzyme for ADPR.

Interestingly, the sirtuin product *O*-acetyl-ADPR, while being a substrate for other Nudix enzymes, is not a substrate for and is only a very weak inhibitor of NUDT9 [24]. On the other hand, *O-*acetyl-ADPR is an agonist for TRPM2, binding to the NUDT9H domain with similar affinity to ADPR [27], indicating that there are important differences between NUDT9 and TRPM2 in terms of substrate recognition and ligand binding. These differences will be of utmost importance when developing specific modulators of either NUDT9 or TRPM2.

In the current work, we synthesized and evaluated novel ADPR analogues with modifications in the terminal ribose to obtain a better understanding of the structural requirements for ligand binding to TRPM2. To identify amino acids interacting with the terminal ribose, we built a homology model of the NUDT9H domain of TRPM2 and computationally docked ADPR. Based on these studies, we chose amino acids in close proximity to, and therefore potentially interacting with, the terminal ribose, prepared TRPM2 point mutants, and evaluated their ability to respond to ADPR.

## Material and Methods

All reagents and solvents were of commercial quality and were used without further purification, unless described otherwise. Unless otherwise stated, all reactions were carried out under an inert atmosphere of argon. ^1^H, ^13^C, and ^31^P NMR spectra were collected on a Bruker 400 MHz or Bruker Avance III 500 MHz spectrometer, and coupling constants are given in Hertz (Hz). High-resolution time-of-flight mass spectra were obtained on a Bruker Daltonics micrOTOF mass spectrometer using electrospray ionization (ES). UV spectra were collected in aqueous solution on a PerkinElmer Lambda EZ 201 or Lambda 3B spectrophotometer. The purity of new tested compounds was determined to be ≥95% by analytical HPLC. HPLC analyses were carried out on a Waters 2695 Alliance module equipped with a Waters 2996 Photodiode Array Detector (210–350 nm). The chromatographic system consisted of a Hichrom Guard Column for HPLC and a Phenomenex Synergi 4µ MAX-RP 80A column (150 × 4.60 mm), eluted at 1 ml/min. Semi-preparative chromatography was performed on a Waters 2525 system equipped with a Waters 2487 Dual λ Absorbance Detector. The system consisted of a Phenomenex Gemini 5E C18 column (250 × 10 mm) eluted at 5 ml/min. Preparative chromatography (RP-18 or Q-Sepharose) was performed on a Pharmacia Biotech Gradifrac system equipped with a peristaltic P-1 Pump and a fixed wavelength UV-1 Optical Unit (280 nm).

### Synthesis of β-methyl-ADP

ADP·Na^+^ (50 mg, 0.111 mmol) was dissolved in 1 ml of MilliQ water and passed through a small Dowex column (50 × 2–100, triethylammonium form). The column was washed with 50 ml of MilliQ and the eluate was evaporated to a thick oil which was dissolved in dry MeOH (6 ml). Dicyclohexylcarbodiimide (360 mg, 1.74 mmol) was added and the mixture was stirred at room temperature for 4 h. Cold water (20 ml) was added and the precipitate formed was filtered. The filtrate was evaporated under reduced pressure, leaving a residue that was purified on an RP-18 column eluted with a gradient of MeCN in 0.05 M TEAB (triethylammonium bicarbonate; 0–30%). The fractions containing the product were collected and evaporated under vacuum; the residue was co-evaporated three times with MeOH to remove excess salt to yield the desired dinucleotide as a triethylammonium salt (33.2 µmol, 30%). ^1^H (400 MHz, D_2_O) *δ* 8.45 (s, 1H, H-2), 8.20 (s, 1H, H-8), 6.08 (br s, 1H, H-1′), 4.48–4.16 (m, 4H, H-3′, H-4′, H-5′), and 3.56 (s, 3H, CH_3_) ppm (note: H-2′ overlapped with the HDO peak); ^31^P (161 MHz, D_2_O) *δ* – 9.6–11.3 (m) ppm; ^13^C (100 MHz, D_2_O) *δ* 155.7 (C-6), 152.9 (C-2, C-8), 122.7 (C-5), 86.9 (C-1′), 84.1 (C-4′), 74.3 (C-2′), 70.5 (C-3′), 65.3 (C-5′), and 53.4 (CH_3_) ppm; HRMS (ES^−^) calcd for C_11_H_16_N_5_O_10_P_2_ 440.1378 (M−H)^−^ found 440.0388.

### Synthesis of β-(tetrahydrofuran-2-yl)methyl-ADP

To a solution of AMP (adenosine 5′-monophosphate) morpholidate (37 mg, 0.084 mmol) and tetrahydrofuan-2-yl methanol monophosphate (17 mg, 0.093 mmol) in 0.2 N MnCl_2_ in formamide (0.5 ml) was added MgSO_4_ (18 mg, 0.169 mmol), and the mixture was stirred at room temperature for 16 h. Precipitation of the product occurred upon the addition of MeCN. Further purification on an RP-18 column eluted with a gradient of MeCN in 0.05 M TEAB (0–30%) afforded after treatment with Chelex 100 the desired dinucleotide as a glassy solid in its sodium form (27 µmol, 32%). ^1^H (400 MHz, D_2_O) *δ* 8.44 (br s, 1H, H-2), 8.20 (s, 1H, H-8), 6.07 (br s, 1H, H-1′), 4.49–4.47 (br, 1H, H-2′), 4.34–4.32 (br, 1H, H-3′), 4.17–4.14 (2H), 3.99–3.97 (1H), 3.86–3.84 (1H), 3.71–3.66 (m, 3H), 3.29–3.28 (1H) (H-4′, 2 × H-5′, THF-CH_2_ and 3 × THF-H), and 1.61–1.35 (m, 4H, 2 × THF-CH_2_) ppm; ^31^P (161 MHz, D_2_O) *δ* – 11.5 (m) ppm; ^13^C (100 MHz, D_2_O) *δ* 158.2 (C-6), 153.0 (C-8), 149.3 (C-4), 140.0 (C-2), 113.3 (C-5), 87.0 (C-1′), 84.0 (C-4′), 79.9 (CH), 74.3 (C-2′), 70.5 (C-3′), 65.2 (C-5′), and 33.4 and 22.7 (both CH_2_) ppm; HRMS (ES^−^) calcd for C_15_H_22_N_5_O_11_P_2_ 510.0790 (M−H)^−^ found 510.0781. UV (H_2_O, pH 7.6) *λ*_max_ 257 nm (*ε* 16 700).

### Synthesis of β-1″-*O*-methyl-ADPR and α-1″-*O*-methyl-ADPR

β-NAD^+^ (10 mg, 15 µmol) was dissolved in MeOH-Na_2_HPO_4_ (0.05 M, aq.) (1:2v/v, 3 ml) and stirred at 60°C. The reaction was monitored by analytical HPLC using an isocratic system of 5% MeCN in 0.1 M TEAB. After 16 h, all starting material was consumed and three new peaks were visible: *R*_t_ = 3.7 min (ADPR), *R*_t_ = 4.3 min (α-1″-*O*-methyl-ADPR), *R*_t_ = 5.3 min (β-1″-*O*-methyl-ADPR). Note that both the anomeric protons of the methyl-ADPR products fall under the HOD peak in the ^1^H-NMR and were revealed by solvent suppression. The crude material was evaporated to dryness, taken up in 0.1 M TEAB (5 ml), and purified by semi-preparative HPLC in the isocratic mode using 0.1 M TEAB–MeCN (95:5, v/v), followed by analysis using analytical HPLC to ensure that only pure fractions were combined. Pure β-1″-*O*-methyl-ADPR (3.4 mg, 5.9 µmol, 39% TEA salt) was obtained as a glassy solid after this step. α-1″-*O*-methyl-ADPR required further purification and was isolated after ion exchange chromatography on Q-sepharose, eluted with a gradient of 0–30% TEAB (1 M) in MilliQ to remove a nicotinamide-related contaminant, followed by re-purification by semi-preparative HPLC. Pure α-1″-*O*-methyl-ADPR (1.5 mg, 2.5 µmol, 17% TEA salt) was obtained as a glassy solid.

β*-*1″*-O*-methyl-ADPR: ^1^H (500 MHz, D_2_O) *δ* 8.40 (s, 1H, H-8), 8.14 (s, 1H, H-2), 6.02 (d, 1H, *J* = 5.8, H-1′), 4.73 (d, 1H, *J* = 1.3, H-1″), 4.65 (dd, 1H, *J* = 5.8, 5.0, H-2′), 4.42 (dd, 1H, *J* = 5.0, 3.5, H-3′), 4.28–4.26 (m, 1H, H-4′), 4.12–4.09 (m, 3H, H-3″, 2 × H-5′), 4.00–3.96 (m, 2H, H-4″, H-5″_a_), 3.88 (dd, 1H, *J* = 4.5, 1.3, H-2″), 3.87–3.83 (m, 1H, H-5″_b_), 3.21 (s, 3H, Me), 3.07 (q, 12H, *J* = 7.5, TEAB CH_2_), 1.15 (t, 18H, *J* = 7.5, TEAB CH_3_) ppm; ^13^C (125 MHz, D_2_O) *δ* 155.6 (C-6), 152.7 (C-8), 149.2 (C-4), 139.8 (C-2), 118.0 (br, C-5), 107.7 (C-1″), 86.7 (C-1′), 83.9 (d, *J* = 9.3, C-4′), 81.2 (d, *J* = 8.5, C-4″), 74.2 (C-2′), 73.9 (C-2″), 70.6 (C-3′), 70.3 (C-3″), 66.4 (d, *J* = 3.9, C-5″), 65.1 (d, *J* = 4.3, C-5′), 55.0 (Me), 46.6 (TEAB CH_2_), 8.2 (TEAB CH_3_) ppm; ^31^P (202 MHz, D_2_O) *δ* – 10.7 (br), −11.4 (br) ppm; HRMS (ES^−^) calcd for C_16_H_24_N_5_O_14_P_2_ 572.0795 (M−H)^−^ found 572.0839; UV (H_2_O, pH 7.6) *λ*_max_ 259 nm (*ε* 15 800).

α-1″-*O*-methyl-ADPR: ^1^H (500 MHz, D_2_O) *δ* 8.38 (s, 1H, H-8), 8.12 (s, 1H, H-2), 6.00 (d, 1H, *J* = 5.9, H-1′), 4.79 (br s, 1H, H-1″), 4.63 (dd, 1H, *J* = 5.9, 4.1, H-2′), 4.44 (dd, 1H, *J* = 4.3, 4.1, H-3′), 4.31–4.28 (m, 1H, H-4′), 4.14–4.12 (m, 2H, 2 × H-5′), 4.10–4.07 (m, 1H, H-4″), 4.04–4.02 (m, 2H, H-2″, H-3″), 3.93–3.90 (m, 2H, 2 × H-5″), 3.22 (s, 3H, Me), 3.04 (q, 9H, *J* = 7.3, TEAB CH_2_), 1.12 (t, 15H, *J* = 7.3, TEAB CH_3_) ppm; ^13^C (125 MHz, D_2_O) *δ* 155.6 (C-6), 152.8 (C-8), 149.1 (C-4), 139.7 (C-2), 124.2 (br, C-5), 103.1 (C-1″), 86.6 (C-1′), 83.8 (d, *J* = 9.3, C-4′), 83.1 (d, *J* = 8.6, C-4″), 74.1 (C-2′), 70.7 (C-2″), 70.3 (C-3′), 69.5 (C-3″), 65.4 (d, *J* = 4.9, C-5″), 65.1 (d, *J* = 5.4, C-5′), 55.3 (Me), 46.5 (TEAB CH_2_), 8.1 (TEAB CH_3_) ppm; ^31^P (202 MHz, D_2_O) *δ –* 11.3 (d, *J* = 21.2), 11.5 (d, *J* = 21.2) ppm; HRMS (ES^−^) calcd for C_16_H_24_N_5_O_14_P_2_ 572.0795 (M−H)^−^ found 572.0787; UV (H_2_O, pH 7.6) *λ*_max_ 259 nm (*ε* 15 800).

### Cell culture

Wild-type HEK293 cells were kept in complete DMEM with Glutamax I (Invitrogen/Life Techonologies, Darmstadt, Germany), 10% (v/v) foetal bovine serum (Biochrom, Berlin, Germany), and 100 U/l pencillin, 100 µg/l streptomycin (Invitrogen/Life Techonologies, Darmstadt, Germany) at 37°C and 5% CO_2_. For HEK293 cells stably expressing TRPM2 (clone #24), G418 sulphate (Biochrom, Berlin, Germany) was added to complete medium (final concentration 400 µg/ml).

### Homology modelling of the Nudix domain of TRPM2

A sequence alignment, based on that of Shen et al. [24], of the TRPM2 Nudix domain with that of NUDT9 is shown in Figure 4. The Sybyl software from Tripos (http://www.tripos.com/) was used to build the model of the Nudix domain of TRPM2. For most of the sequence, the 1QVJ crystal structure of NUDT9 was mutated, one residue at a time, to have the TRPM2 sequence. For the three regions with deletions or insertions (those areas in pink in Figure 4), the loop search functionality in the software was used to search for suitable conformations. To accommodate the deletion of residues Ser 192 and Gly 193 from the NUDT9 sequence, residues Pro 190 to His 195 were deleted and replaced with the SIKK sequence of TRPM2. The residues from Ser 238 to Leu 262 in the NUDT9 sequence were deleted and replaced with the FENLLKCGM sequence from TRPM2. The residues from Met 295 to Leu 298 from the NUDT9 sequence were deleted and replaced with the VELNRLNS sequence of TRPM2. These three regions were then individually each put through a short minimization procedure prior to the whole protein being minimized.

### Modelling ADPR into the TRPM2 Nudix domain

There are two approaches to positioning ADPR in the binding site of the TRPM2 Nudix domain. The first is to search for crystal structures that have similar secondary and tertiary structure to that of the TRPM2 Nudix domain and that have ADPR in the binding site. These structures can then be superimposed on the TRPM2 Nudix domain and the ADPR copied from one molecule into the other. The second is to dock ADPR into the binding site computationally.

There are 50 structures in the PDB that have ADPR bound to them. However, for only five of these proteins is the secondary and tertiary structure sufficiently similar to that of the TRPM2 Nudix domain to allow the structures to be superimposed. These five proteins, all from bacterial species, are listed in Supplementary Table S1. Superimposing these five structures on each other revealed that all have a similar 3D structure in the ADPR-binding domain, but that 2QJO has an additional α-helical domain. All five structures have the phosphates and the two ribose moieties in similar positions, but the orientation of the adenine ring in 1G9Q [20], 1MK1 [21], and 1V8L [22] differs from that in the 2QJO [26] and 3GZ8 [28]. Consequently, 1G9Q and 2QJO were used as templates for modelling ADPR into the TRPM2 Nudix domain. The lack of sequence similarity between the proteins did not allow a sequence alignment, but DALI (http://ekhidna.biocenter.helsinki.fi/dali_lite/start) was used to align the 1G9Q and 2QJO structures to the TRPM2 Nudix domain structure based on their structural similarity. The C-terminal parts of the structures are fairly similar, but there is little structural similarity in the N-terminal parts. The ADPR was copied from the template molecules into the TRPM2 Nudix domain and the structures put through brief minimization procedures. Between the two structures, there is very little difference in the positions of the backbone atoms, but there are some small differences in the positions of the side chain atoms. GOLD (http://www.ccdc.cam.ac.uk/products/life_sciences/gold/) was used to dock ADPR computationally into the TRPM2 Nudix domain. The homology model with the 1G9Q ADPR in the binding site was used as the starting point. An ADPR model was built using the Schrödinger software (http://www.schrodinger.com/) and was docked (25 times) with the requirement that the ligand centroid be within 5 Å of the centroid of the 1G9Q ADPR molecule. Although the docked poses duplicated neither the 1G9Q nor the 2QJO crystal structure poses of ADPR, the poses that were observed were done so multiple times.

### Generation of stable cell line

The generation of a HEK293 clone stably expressing TRPM2 (clone #24) has been described previously [25].

### Patch-clamp experiments

Cells were seeded at low density to 35 mm cell culture dishes (Greiner BioOne, Frickenhausen, Germany). Before the experiment, culture medium was replaced with bath solution [140 mM NMDG, 3.3 mM MgCl_2_, 1 mM CaCl_2_, 5 mM KCl, 10 mM HEPES (pH 7.4) (HCl), 5 mM glucose]. Patch pipettes with a resistance between 1.3 and 4.5 MΩ were pulled from 1.5 mm borosilicate glass (Science Products, Hofheim, Germany) using a Model P-97 horizontal micropipette puller (Sutter Instruments, Novato, U.S.A.) and filled with intracellular solution (120 mM KCl, 8 mM NaCl, 1 mM MgCl_2_, 10 mM HEPES (pH 7.2) (KOH), 10 mM EGTA, and 5.6 mM CaCl_2_) resulting in a free [Ca^2+^] of 0.2 µM as calculated with the CaBuf program (G. Droogmans, KU Leuven formerly available under ftp://ftp.cc.kuleuven.ac.be/pub/droogmans/cabuf.zip). To test for agonist activity, the test compound (final concentration 100 µM) was included in the pipette solution, whereas during tests for antagonist activity the pipette solution contained a combination of the test compound (900 µM) and ADPR (100 µM). Currents were recorded in the whole-cell configuration [29] using an EPC10 amplifier and PatchMaster software (HEKA Elektronik, Lamprecht, Germany). Cells were held at a potential of −50 mV. After break-in, a voltage ramp from −85 to 20 mV in 140 ms was applied every 5 s for a total of 450 s. The patch-clamp amplifier was set to compensate for 70% of the series resistance. Recordings were further analyzed when the series resistance did not increase to more than 10 MΩ during the experiment and the maximum current did not exceed 20 nA (at which the amplifier started to clip).

### Mutagenesis

Mutations T1347V, Y1349F, L1381I, R1433M, and Y1485F were introduced into pIRES2-EGFP-TRPM2 by the use of the QuikChange Site-Directed Mutagenesis Kit (Agilent Technologies, Waldbronn, Germany) using appropriate primer pairs. The presence of the mutation in the resulting plasmids was confirmed by sequencing the targeted region. For constructs showing reduced calcium signals, the complete open reading frame for TRPM2 was sequenced (Eurofins MWG Operon, Ebersberg, Germany). Primers used for mutagenesis and sequencing are included in Supplementary Table S2.

### Transient transfection of HEK293 cells for Ca^2+^ imaging

Wild-type HEK293 cells were detached using Trypsin–EDTA (Invitrogen/Life Technologies, Darmstadt, Germany), and the cell density was adjusted to 1 × 10^6^ cells in 3 ml of complete DMEM. The transfection complex was prepared by combining either 5 µg of pIRES2-hTRPM2wt or 5 µg of one of the vectors modified by QuikChange mutagenesis and 10 µl of jetPEI reagent (PolyPlus Transfection, Strasbourg, France) in a total volume of 500 µl of 150 mM NaCl and incubating for 45 min at room temperature. The transfection complex was added to the cell suspension. After mixing by inversion, cells were seeded to 35 mm glass bottom culture dishes (MatTek, Ashland, U.S.A.) and kept at 37°C and 5% CO_2_ overnight.

### Ca^2+^ imaging

One day post transfection, cells were loaded with Fura2 by adding Fura2/AM (Merck/Calbiochem, Darmstadt, Germany) to the medium to a final concentration of 4 µM and incubated for 30 min at 37°C/5% CO_2_. After loading, the medium was carefully replaced by 1 ml of extracellular solution [140 mM NaCl, 5 mM KCl, 1 mM MgCl_2_, 1.8 mM CaCl_2_, 10 mM glucose, 0.1% BSA, and 15 mM HEPES (pH 7.4)] pre-warmed to 37°C. Loaded cells were placed on the stage of one of two imaging systems (PerkinElmer, Rodgau, Germany) consisting of an inverted fluorescence microscope (either DM-IRE2 or DM-IRBE, Leica, Solms, Germany) with a 40× objective (NA 0.5–1.0), a light source with tunable excitation wavelength (either Polychrome II or Polychrome IV, Till Photonics, Gräfelfing, Germany), and a CCD camera (either C4742-95-12ER or C4742-95-12NRB, Hamamatsu, Enfield, U.K.) controlled by a computer (G5 Mac, Apple computer) running the OpenLab v4.04 software (PerkinElmer, Rodgau, Germany). Before the start of ratiometric calcium imaging, an image of EGFP fluorescence (excitation at 488 nm) of the cell field was taken to restrict further analysis to transfected cells. The changes in Fura2 fluorescence were analyzed by taking image pairs (excitation at 340 and 380 nm) every 10 s and by online calculation of ratio images. After five ratio images (∼50 s), hydrogen peroxide (Sigma–Aldrich) was added to a final concentration of 1 mM. Regions of interest including single-transfected cells (as identified by EGFP fluorescence) were analyzed for changes in the ratio over time. For quantitative analysis, the maximum ratio for each cell was determined. The number of cells above the threshold ratio was determined using MS Excel 2003 (Microsoft Corporation, Redmond, U.S.A.)

### Biotinylation of cell surface proteins

Wild-type HEK293 cells (3 × 10^5^) were seeded into T25 cell culture flasks and kept at 37°C and 5% CO_2_. After 3 days, cells were transfected with either 7.5 µg of pIRES2-EGFP, pIRES2-EGFP-hTRPM2wt [30] or 7.5 µg of one of the vectors modified by QuikChange mutagenesis using Lipofectamine LTX with PLUS reagent (Invitrogen) according to the manufacturer's protocol. Transfection was confirmed 24 h after transfection by EGFP expression. At 48 h post transfection, cells were washed with D-PBS (with Ca^2+^ and Mg^2+^) and incubated for 30 min at room temperature with 1 mg/ml EZ-Link Sulfo-NHS-LC-Biotin (Pierce/Thermo Scientific). After detachment and collection of the labeled cells, membrane proteins were isolated using the ProteoExtract Native Membrane Protein Extraction Kit (EMD Millipore) according to the manufacturer's protocol. Protein concentration was determined using the Bio-Rad protein assay (Bio-Rad) in 96-well microplates. For pulldown of biotinylated proteins, 50 µl of NeutraVidin Agarose Beads (50% aqueous slurry, Pierce/Thermo Scientific) and 400 µg of the membrane proteins were incubated overnight using overhead rotation. Total membrane protein samples (10 μg each) and biotinylated protein sample after pulldown (from 400 µg of membrane protein) were separated by 7.5% SDS–PAGE and transferred onto PVDF membranes. A pre-stained marker was used, so that membranes could be cut in half between 100 and 150 kDa. The upper part of the membrane was probed for TRPM2 by incubation with anti-TRPM2 antibody (Novus) and HRP-conjugated goat anti-rabbit IgG (Dianova) secondary antibody, whereas the lower part of the membrane was probed for Na^+^/K^+^-ATPase by incubation with a mouse anti-Na^+^/K^+^-ATPase α1 antibody (Abcam) and HRP-conjugated goat anti-mouse IgG (Dianova) secondary antibody. Membranes were incubated for 5 min with a 1:10 mixture of SuperSignal West Dura chemiluminescent substrate (Pierce/Thermo Scientific) and SuperSignal West Pico chemiluminescent substrate (Pierce/Thermo Scientific), and chemiluminescence was detected using a LAS-3000 Intelligent Dark Box (Fujifilm, Tokyo, Japan).

### Statistics and graphs

Graphs were generated using GraphPad Prism v6.03 and SigmaPlot10.0 (Systat Software, San Jose, U.S.A.). If not indicated otherwise, all data are represented as mean values ± standard error of the mean. For statistical testing, GraphPad Prism v6.03 and SigmaStat3.5 (Systat Software, San Jose, U.S.A.) were used except for the *χ*^2^ test for which R (version 2.14.2, www.r-project.org/) was used. If not indicated otherwise, a significance level (*α*) of 0.05 wa*s* adopted*.*

## Results

### Preparation of ADPR analogues with modifications to the terminal ribose structure

**Figure 1.**
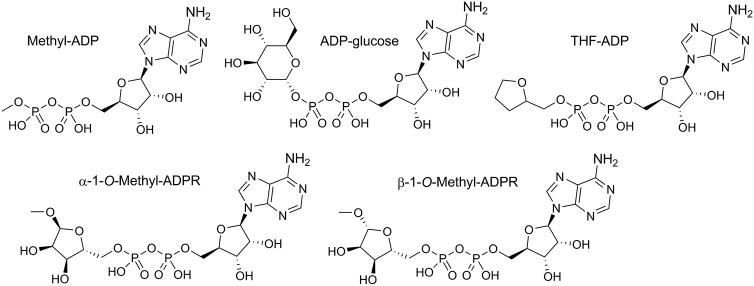
ADPR analogues modified in the terminal ribose Chemical structures of ADPR derivatives with modifications in the terminal ribose.

To investigate the role of the terminal ribose of ADPR in the activation of TRPM2, we synthesized four derivatives of ADPR (Figure 1). The sodium salt of ADP was converted into the triethylammonium form and coupled with methanol to generate β-methyl-ADP, an analogue with a much smaller terminal substituent than ribose. AMP-morpholidate was coupled to tetrahydrofuran-2-yl methanol monophosphate to prepare β-(tetrahydrofuran-2-yl)methyl-ADP (THF-ADP). The THF substituent retains the five-membered heterocyclic ring, but lacks hydroxyl groups that are potentially involved in hydrogen bonding to the protein. By hydrolysis of β-NAD^+^ in methanol [31], we prepared and separated both α-1″-*O*-methyl-ADPR and β-1″-*O*-methyl-ADPR. Methylation of the anomeric hydroxyl group prevents interconversion between the α- and β-forms of the terminal ribose. In addition to our synthetic analogues, we used commercially available ADP-glucose as a derivative with a bulkier terminal substituent that contains hydroxyl groups and thus could potentially engage in hydrogen bonding (Figure 1). For schemes showing preparation of synthetic analogues, see Supplementary Figures S1–S3.

### Both terminal moieties of ADPR are required for agonist activity

**Figure 2.**
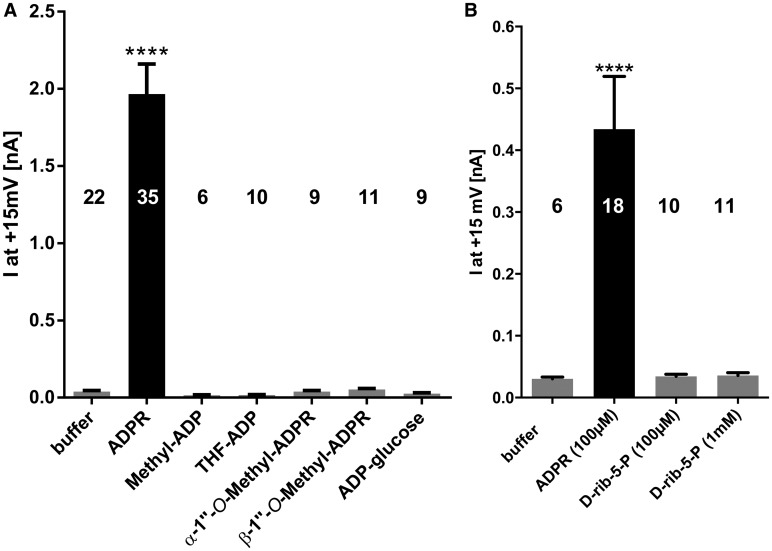
Both terminal moities of ADPR are essential for agonist activity. Pipette was filled with either buffer, buffer with 100 µM ADPR, or buffer with 100 µM of the indicated ADPR derivative. Channel activation in the whole-cell mode was followed by applying repetitive voltage ramps from −85 to +20 mV every 5 s. Maximum currents at +15 mV during the experiment (450 s) were determined from the voltage ramps and are represented as mean ± SEM. Significant differences versus buffer control are indicated by asterisks (****P ≤ 0.0001, one-way ANOVA with *post hoc* comparison using Bonferroni correction). Because during some experimental sessions data were acquired for both data sets in this figure and Figure 3, the data in the control condition (100 µM ADPR) partially overlap. Numbers on top of the bars indicate the number of cells analyzed. d-rib-5-P was tested in a different series of experiments and thus is included as (**B**).

The activity of the ADPR derivatives in Figure 1 at the TRPM2 channel was experimentally determined in HEK293 cells stably expressing wild-type human TRPM2. Since, under the chosen ionic conditions for the whole-cell patch-clamp experiments, outward currents were more pronounced than inward currents, the net outward current was chosen as a readout for the evaluation of the ADPR derivatives. Using a pipette solution containing 100 μM ADPR (and 0.2 µM free Ca^2+^), robust TRPM2 channel activation was observed roughly 100 s after break-in. Under these conditions, none of the analogues in which the terminal ribose was replaced activated the channel (Figure 2A). Likewise, the fragment d-rib-5-P, to mimic the terminal ribose region of ADPR, also did not activate TRPM2, even at a concentration of 1 mM (Figure 2B).

### ADPR analogues with the terminal ribose replaced by small substituents can antagonize activation of TRPM2 by ADPR

**Figure 3.**
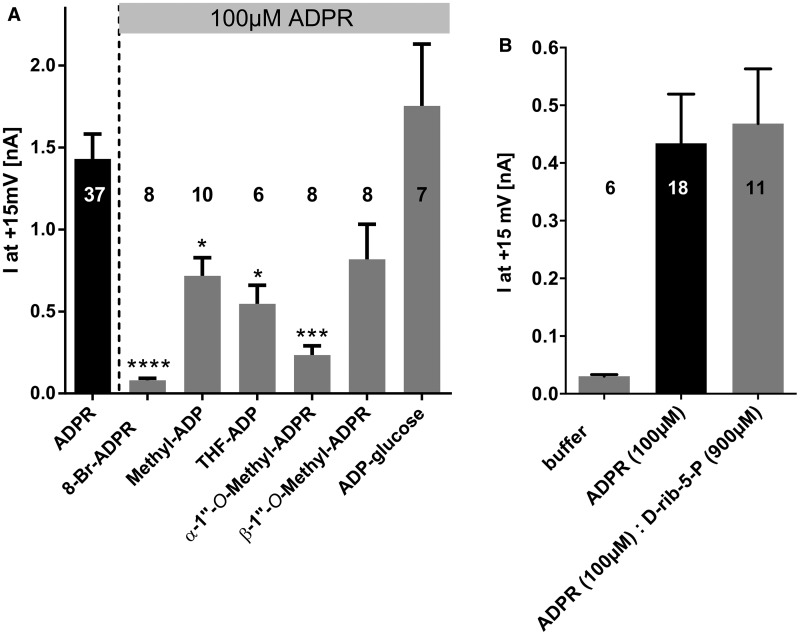
ADPR analogues with the terminal ribose replaced by small substituents antagonize activation of TRPM2 by ADPR. Pipette solution contained either only 100 µM ADPR or 100 µM ADPR combined with 900 µM of the indicated ADPR derivative. Peak currents at +15 mV obtained from repetitive voltage ramps were determined from the voltage ramps and are represented as mean ± SEM. Significant differences versus control (100 µM ADPR) are indicated by asterisks (*P ≤ 0.05, ***P ≤ 0.001, ****P ≤ 0.0001, one-way ANOVA with *post hoc* comparison using Bonferroni correction). Numbers on top of the bars indicate the number of cells analyzed. d-rib-5-P was tested in a different series of experiments and thus is included as (**B**).

We tested whether our ADPR analogues could antagonize activation of TRPM2 by ADPR by combining 100 μM ADPR with an excess of an analogue (900 μM) in the pipette solution (0.2 µM free Ca^2+^) and observing the effect on the net outward current (Figure 3A). While the published analogue 8-Br-ADPR [32] inhibited 95% of the net outward current evoked by ADPR alone, ADP-glucose did not interfere with TRPM2 activation. Interestingly, there was a significant partial inhibition by methyl-ADP (50% inhibition of the current evoked by ADPR alone), THF-ADP (62% inhibition), and α-1″-*O*-methyl-ADPR (84% inhibition). While not significant, there was also a trend to lower currents with β-1″-*O*-methyl-ADPR (43% inhibition). In contrast, d-rib-5-P alone did not inhibit the activation of TRPM2 by ADPR (Figure 3B). These data suggest that the four analogues with small or ribose-like terminal substituents bind to the NUDT9H domain of TRPM2 and, when applied in excess, are able to compete with ADPR, while neither an analogue with a larger substituent replacing the terminal ribose (glucose) nor a simple fragment (d-rib-5-P) can compete with ADPR.

**Figure 4.**
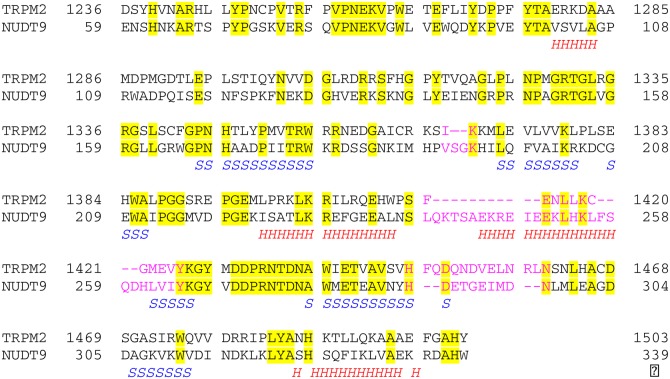
Sequence alignment of human TRPM2 with human NUDT9. Taken from [24]. Identical residues are shown highlighted in yellow. Areas of significant difference, i.e. deletions from or insertions into one sequence relative to the other, are shown in pink text. From the crystal structure of human NUDT9 [24] (PDB code 1QVJ), areas of helix (H) and sheet (S) as identified by PyMol (DeLano Scientific LLC; http://www.delanoscientific.com/) are shown. NUDT9 sequence taken from UniProtKB entry Q9BW91. TRPM2 sequence taken from UniProtKB entry O94759.

### Abolishing the hydrogen bonding from Arg1433 and Tyr1349 interferes with activation of TRPM2 by ADPR

**Figure 5.**
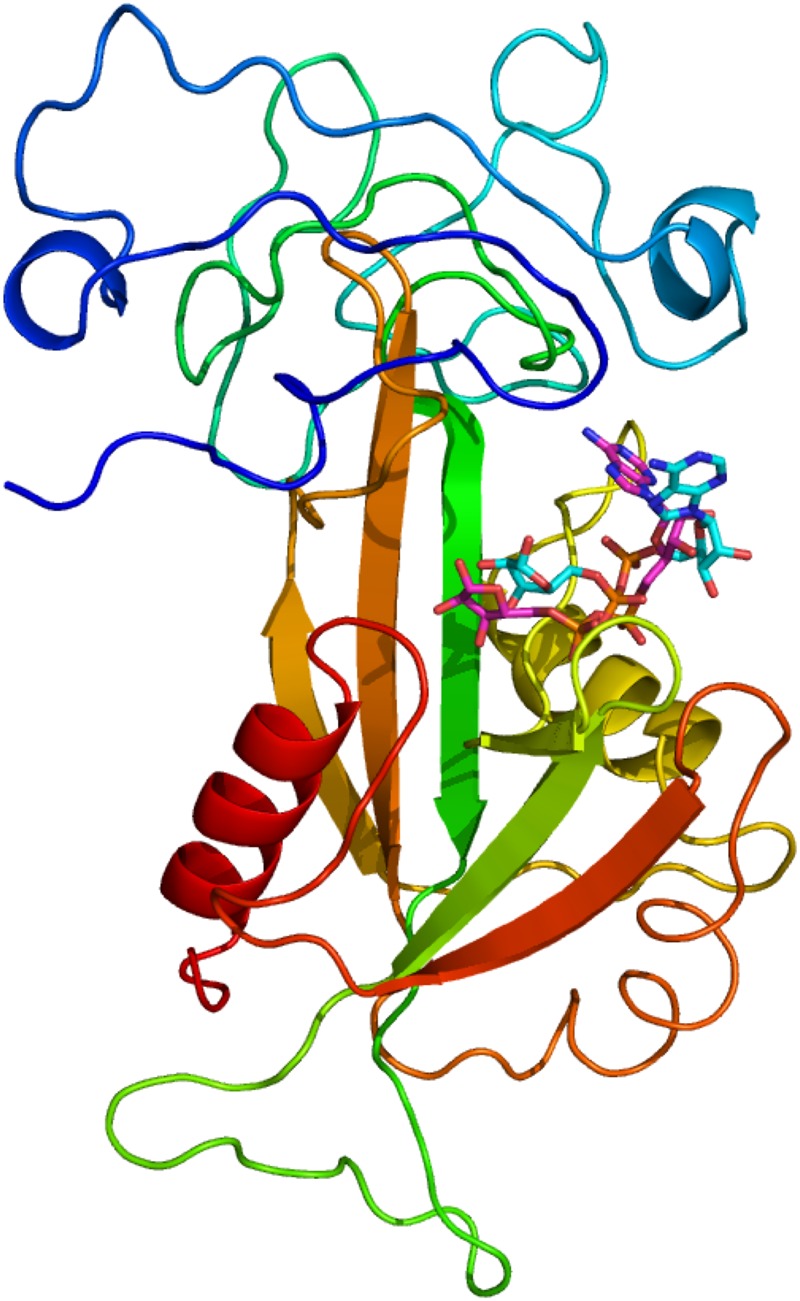
The homology modelled structure of the TRPM2 NUDIX domain. The protein is shown coloured blue at the N-terminus through to red at the C-terminus. The cyan ligand is ADPR copied into the TRPM2-binding site from the 1G9Q crystal structure. The purple ligand is ADPR copied into the TRPM2-binding site from the 2QJO crystal structure.

**Figure 6.**
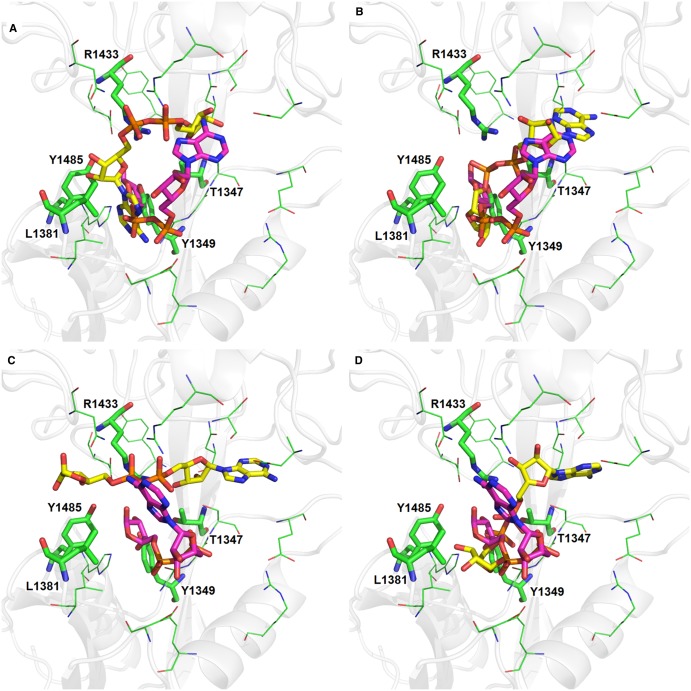
The ADPR-binding site. The protein secondary structure is shown as the transparent grey cartoon. Selected residues forming the binding site are shown in green: residues described in the text as being mutated are shown as sticks and are labelled. The four molecules of ADPR shown in yellow were docked into the binding site using GOLD. The ligand in purple is copied into the binding site from the 2QJO crystal structure (**A** and **B**) and the 1G9Q (**C** and **D**).

**Figure 7.**
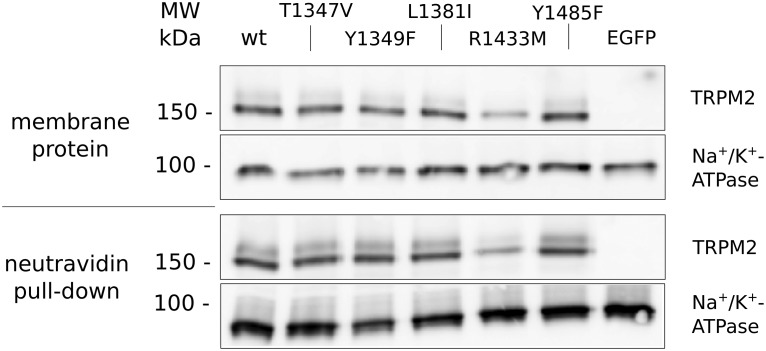
The point mutations T1347, Y1349, L1381I, R1433, and Y1485 do not interfere with expression of human TRPM2 in the plasma membrane. HEK293 cells were transiently transfected with an expression vector for EGFP and either hTRPM2 wild-type (wt) or a channel carrying the indicated mutation. At 48 h post transfection, cells were surface-biotinylated using a non-permeant biotinylation reagent and membrane proteins were isolated from the cells. Membrane protein (400 µg) was incubated with neutravidine agarose and biotinylated proteins were isolated. On one 7.5% SDS–PAGE, 10 µg of total membrane protein for each condition was separated on another 7.5% SDS–PAGE, the complete supernatant from the pulldown for each condition. Both were blotted on PVDF membrane and split in half between the marker bands for 100 and 150 kDa. The lower parts were probed for Na^+^/K^+^-ATPase and the upper parts for TRPM2. Since there were wide differences in signal intensity between TRPM2 and Na^+^/K^+^-ATPase, on the one hand, and total membrane protein and pulldown, on the other, different exposure times were applied for the four strips of western blot depicted. All mutant forms of TRPM2 can be detected in the plasma membrane.

The consequential next step was to look for molecular interaction partners of the terminal ribose. We started out by sequence comparison of TRPM2 and NUDT9 (Figure 4) and subsequently building a homology model of the NUDT9H domain of TRPM2 (Figure 5). To see how ADPR might bind to TRPM2, the crystal structures of two structurally homologous proteins with ADPR in the binding site were superimposed on the homology model and the ligand copied into the TRPM2-binding site. The two ADPR molecules have quite different conformations (Figure 6). To examine further possible ADPR-binding modes, ADPR was computationally docked into the binding site. The dockings produced a range of binding poses but failed to reproduce the crystal structure poses (Figure 6). ADPR is a large, highly flexible molecule and, combined with the wide and open binding site of TRPM2, we were not able to determine a definitive pose for ADPR in the TRPM2-binding site. Examination of the homology model with the various docked and modelled poses indicated that the amino acid residues Thr1347, Tyr1349, Leu1381, Arg1433, and Tyr1485 will be in proximity to the ligand and therefore may interact with the terminal ribose of ADPR. To determine the role of these amino acid residues experimentally, we introduced point mutations at the respective positions in the TRPM2 expression vector. While the mutations were deliberately chosen to be conservative, initially we could not exclude any misfolding, degradation, or lack of trafficking of the channel to the plasma membrane. We therefore checked expression at the cell surface in transiently transfected cells by biotinylation and pulldown of surface proteins. As shown in Figure 7, all of the mutants were detectable in the plasma membrane of the transfected cells, while the channel was not detectable in membranes of cells transfected with the vector backbone (pIRES2-EGFP). Since carrying out this work, Yu et al. [33] proposed identification of not only the binding pocket of ADPR in the NUDT9H domain of TRPM2 based on induced docking and molecular dynamics simulation, but also a specific binding pose of the ligand. The authors of this study identified amino acid residues important for specific interactions with ADPR by their docking studies and confirmed them by patch-clamp analysis. Our approach to homology modelling/docking is a more cautious one: due to the limits of predicting side chain orientation by homology modelling and the huge conformational freedom of ADPR, we are sceptical that it would be possible to reliably identify a specific pose of ADPR within the binding pocket of the NUDT9H domain.

**Figure 8.**
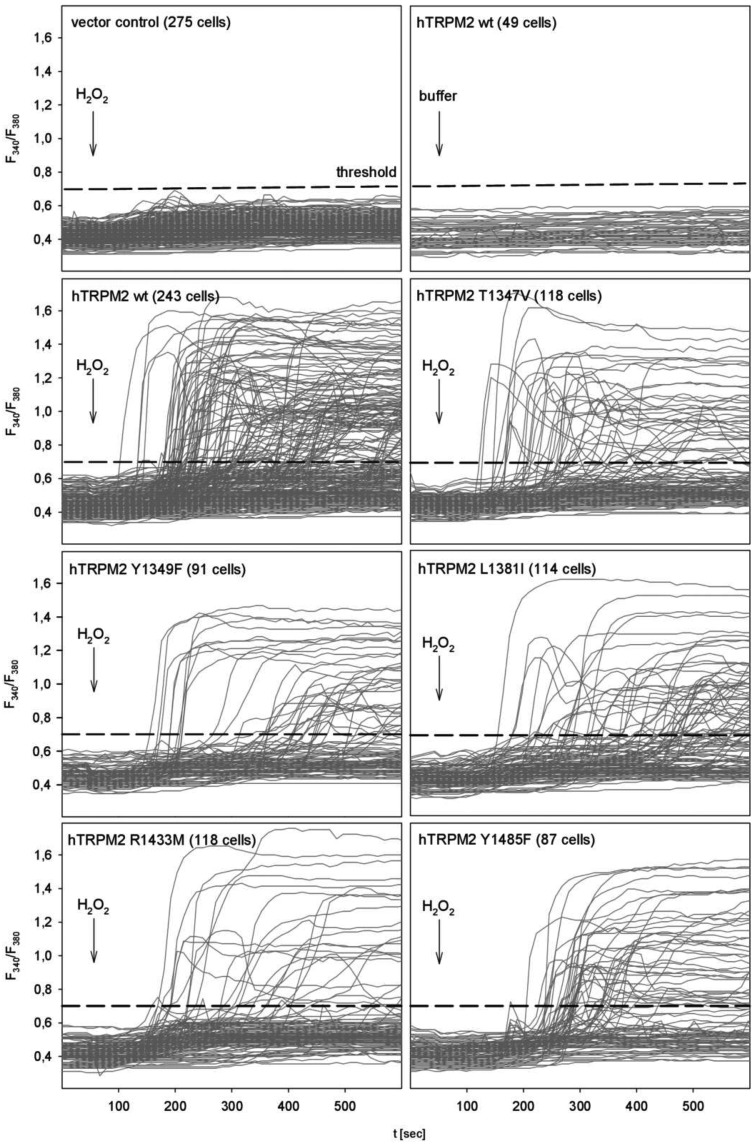
Impact of mutations on the hydrogen peroxide-mediated TRPM2 activation. HEK293 cells were transiently transfected with an expression vector for EGFP and either human wild-type TRPM2 or a channel carrying the indicated mutation. At 24 h post transfection, cells were loaded with Fura2 and subjected to calcium imaging. At the indicated time, either buffer or hydrogen peroxide (final concentration 1 mM) was added. Analysis was restricted to EGFP-positive cells. For differentiation between responding and non-responding cells, the indicated threshold of 0.7 was chosen.

To investigate the effect of the point mutations on TRPM2, transiently transfected HEK293 cells were stimulated by H_2_O_2,_ and [Ca^2+^]_i_ was analyzed by single cell Ca^2+^ imaging. H_2_O_2_ is thought to activate PARP and PARG, resulting in an increase in intracellular ADPR which in turn activates TRPM2 [15]. The lack of response to H_2_O_2_ in HEK293 cells transfected with the vector backbone (pIRES2-EGFP) and to buffer addition in HEK293 cells transfected with the wild-type of human TRPM2 demonstrates the specificity of this approach (Figure 8, upper most panels).

**Figure 9.**
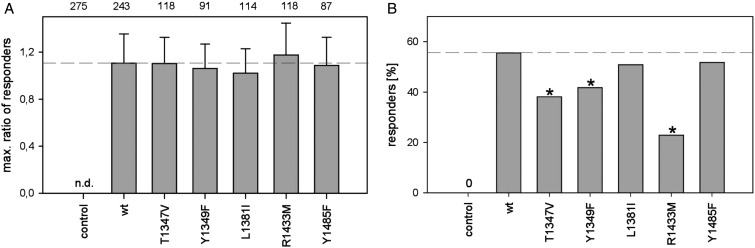
R1433 and, to a lesser extent, T1347 and Y1349 are involved in the activation of TRPM2 by ADPR. (**A**) Maximum ratio achieved by responding cells after stimulation with H_2_O_2_ (dashed line indicates the level of positive control, indicated as mean ± SEM, no significant difference when tested using ANOVA). (**B**) Fraction of cells responding to H_2_O_2_ stimulation with a ratio above the threshold (see Figure 5) during the experiment (dashed line indicates the level of positive control, *indicates significant difference in distribution compared with hTRPM2wt, Pearson's *χ*^2^ test with Yates' continuity correction, *P* < 0.05).

Since, even in cells transfected with the expression vector for wild-type TRPM2, not all of EGFP positive, and thus successfully transfected, cells responded to H_2_O_2,_ we chose a Fura2 threshold ratio of 0.7 that was higher than all tracings of both negative control groups (Figure 8, uppermost panels) to differentiate between responding and non-responding cells. Comparison of the mean amplitude of responders for cells transfected with the expression vectors for wild-type TRPM2 and any TRPM2 mutant showed no significant difference (Figure 9A). This is most probably due to the all-or-nothing nature of channel activation as a result of the positive feedback by calcium [18].

In contrast, when looking at the fraction of responders, different effects were observed for the different point mutations in the NUDT9H domain (Figure 9B). The addition of H_2_O_2_ to cells expressing wild-type TRPM2 defines the upper limit (Figure 8, upper row) with ∼55% responders_._ The strongest effect was observed with Arg1433 → Met. Replacement of arginine, with its potential to form hydrogen bonds, by methionine, which is similar in bulk but not able to form hydrogen bonds, resulted in a 59% reduction in responding cells. Replacing another potential hydrogen bond donor by exchanging Tyr1349 for Phe or increasing the hydrophobicity of the binding pocket by replacing Thr1347 with Val also resulted in significant, although less pronounced, reductions in the number of responding cells (25 and 31% reduction, respectively). On the other hand, decreasing the size of the binding pocket by replacement of Leu1381 with the bulkier Ile or abolishment of the hydrogen bonding potential of Tyr1485 by replacing it with Phe had no significant impact on the fraction of responders.

## Discussion

Our data show that all of our ADPR analogues with modifications to the terminal ribose lack the ability to activate TRPM2. ADP-glucose and the two 1″-*O*-methyl-ADPR analogues may conceivably no longer fit inside the TRPM2-binding pocket. However, the two derivatives, β-methyl-ADP and THF-ADP, with terminal substituents that require less space than d-ribose should fit into the binding pocket. Therefore, this result suggests that either the binding energy is too low and the affinity is too small, or that these compounds, while binding, lack the ability to engage in specific interactions required for activation of the channel. In the latter case, the derivatives would bind and act as competitive inhibitors interfering with activation of the channel by ADPR. We therefore determined whether the ADPR derivatives inhibit activation of the channel by ADPR. Four of our compounds act as antagonists, with substituents that have increasing shape similarity to the terminal ribose showing improved efficacy (Figure 3). This suggests that the complete terminal ribose of ADPR is not required for binding to the NUDT9H domain in terms of affinity. ADP-glucose with multiple H-bonding potential, but a terminal substituent larger than d-ribose, does not inhibit the channel, presumably because it does not fit into the binding pocket sterically. ADP has previously been shown to neither activate TRPM2 (at 100 µM [4]) nor inhibit activation of TRPM2 by ADPR (at 900 µM [25]), and thus obviously does not bind to the NUDT9H domain. Small substituents at the beta-phosphate can apparently partially compensate for this lack in affinity due to the absence of the d-ribose, probably as a result of the hydrophobic effect. While methyl-ADP and THF-ADP apparently bind to TRPM2, both lack the ability to activate the channel, indicating that the terminal ribose in ADPR does engage in some specific interaction necessary for the gating of the channel that cannot easily be mimicked, for instance, by the five-membered heterocyclic ring of THF-ADP alone. Both α- and β-1″-*O*-methyl-ADPR were also somewhat unexpectedly unable to activate the channel at 100 µmol/L, but showed antagonist activity at TRPM2. This highlights the sensitivity of the terminal ribose-binding pocket to even minor changes and probes, for the first time, whether the NUDT9H domain of TRPM2 is able to differentiate between the two anomeric forms of ADPR which are in equilibrium in solution and present in roughly equal amounts (α:β = 55:45 in D_2_O as determined by ^1^H NMR [34]). Owing to the fast equilibrium of the free glycoside in ADPR, the two anomers cannot be individually isolated. The methyl glycosides of ADPR are the smallest possible glycosides, and methylation prevents anomer interconversion in solution, allowing compounds with discrete α- and β-configurations at the 1″-position to be obtained in isolation. Interestingly, the α-anomer gave a significantly higher reduction in the whole-cell current. Our novel analogues of the terminal ribose, in particular α- and β-1″-*O*-methyl-ADPR, clearly demonstrate that masking the hydroxyl group at the anomeric C1″ deletes all agonist activity. This suggests, for the first time, that the C1″-hydroxyl group plays a pivotal role in ADPR activation of the TRPM2 channel. The effect of the configuration at the anomeric position will be explored in more detail in future studies.

Using a homology model built on the basis of the crystal structure of human NUDT9 with different docked and modelled poses of ADPR in the binding pocket, we identified Thr1347, Tyr1349, Leu1381, Arg1433, and Tyr1485 as amino acids in proximity to the terminal ribose of ADPR that might potentially engage in hydrogen bonding or otherwise interact with the natural ligand. The fact that the fraction of responding cells is significantly reduced after abrogating the hydrogen bonding potential by mutation of Arg1433, Tyr1349, and Thr1347 indicates that the side chains of these amino acids are most probably involved in the molecular interaction between the NUDT9H domain of TRPM2 and ADPR, in particular with its terminal ribose. The critical involvement of Arg1433 and Tyr1349 in ADPR binding is consistent with the structurally similar, bifunctional (NMNAT)/ADPR pyrophosphatase from the cyanobacterium *Synechocystis* sp. [26]. Further, three amino acid residues, Arg1433, Thr1347, and Tyr1349, were identified as binding partners of ADPR both in our work and in a recent study on TRPM2 [33]. However, while our docking studies indicate that Arg1433 might hydrogen bond with the terminal ribose of ADPR, electrostatic interaction between Arg1433 and the pyrophosphate backbone was proposed by Yu et al. [33]. Since the pyrophosphate and terminal ribose are directly connected, and thus in close proximity, the differential interpretation of the experimental data might result from the high flexibility of ADPR allowing for multiple potential poses within the binding site. Similarly, we would place Thr1347 close to the terminal ribose and hydrogen bonding with it, since loss of the HO-group in Thr1347 → Val decreased the percentage of responding cells (Figure 9B). In contrast, Yu et al. [33] propose electrostatic interaction between Thr1347 and the pyrophosphate backbone. Finally, the fact that the Tyr1349 → Phe results in a reduction in responding cells indicates, in our view, an interaction between Tyr1349 and the terminal ribose by hydrogen bonding, while electrostatic interactions between the π-electrons of the phenyl moiety of tyrosine with the adenine base of ADPR were predicted in [33]. Other residues found by Yu et al. [33], e.g. H1488 or E1409, were not predicted by our docking studies.

## Conclusions

The inability of our ADPR analogues modified in the terminal ribose to activate TRPM2 highlights the structural importance of this region, and specifically of the C1″-hydroxyl group for opening the channel. Our complementary results using point mutations to abrogate individual hydrogen bonds proposed by a homology model of human NUDT9 are supportive of the proposed ADPR-binding mode and give insights into the critical residues for channel activation and the possible mode of ADPR binding. This combined molecular and chemical biology approach is an important step towards understanding the structural basis of the gating mechanism and will also facilitate the rational design of second-generation novel TRPM2 inhibitors/ADPR antagonists.

## Abbreviations

ADP: adenosine 5′-diphosphate
ADPR: adenosine 5′-diphosphoribose
AMP: adenosine 5′-monophosphate
d-rib-5-P: d-ribose 5-phosphate
NAD^+^: nicotinamide adenosine 5′-dinucleotide
NMDG: N-methyl-D-glucamine
NMNAT: nicotinamide mononucleotide adenylyltransferase
NUDT9H: NUDT9 homology domain
PARP: poly(ADPR) polymerase
PARG: poly(ADPR) glycohydrolase
PKC: protein kinase C
TEA: triethylammonium
TEAB: triethylammonium bicarbonate
TRP: transient receptor potential channels
TRPM2: transient receptor potential channel, subfamily melastatin, member 2
